# Predicting Biochemical Disease-Free Survival after Prostate Stereotactic Body Radiotherapy: Risk-Stratification and Patterns of Failure

**DOI:** 10.3389/fonc.2016.00168

**Published:** 2016-07-08

**Authors:** Alan Katz, Silvia C. Formenti, Josephine Kang

**Affiliations:** ^1^Flushing Radiation Oncology Services, New York, NY, USA; ^2^Department of Radiation Oncology, Weill Cornell Medical College, New York, NY, USA

**Keywords:** prostate cancer, stereotactic body radiotherapy, Gleason score, prostate-specific antigen

## Abstract

**Background and purpose:**

To determine appropriate risk-stratification factors for prostate cancer patients undergoing stereotactic body radiotherapy (SBRT).

**Materials and methods:**

Between 2006 and 2010, 515 patients with organ-confined prostate cancer were treated with a regimen of five-fraction SBRT to dose of 35–36.25 Gy. By NCCN criteria, 324 patients were low risk, 153 were intermediate risk, and 38 were high risk. Patients were defined as unfavorable intermediate risk if Gleason 4 + 3 = 7 or >1 intermediate-risk factors (cT2b, c, PSA 10–20, Gleason 3 + 4 = 7). Cox regression analysis was used to determine risk factors significantly associated biochemical failure, and patterns of failure analyzed.

**Results:**

With median follow-up of 84 months, the 8-year disease-free survival was 93.6, 84.3, and 65.0% for low, intermediate, and high-risk group patients, respectively. Based on the above definition, 106 favorable intermediate-risk patients had excellent outcomes, with no significant difference compared to low-risk patients (7-year DFS 95.2 vs. 93.2%, respectively). The 47 unfavorable intermediate-risk patients had worse outcomes, similar to high-risk patients (7-year DFS 68.2 vs. 65.0%, respectively). Gleason score was the only significant factor associated with biochemical failure on multivariate analysis (*p* = 0.0003).

**Conclusion:**

Patients with favorable intermediate-risk disease have excellent outcomes, comparable to low-risk patients. Patients with unfavorable intermediate-risk disease have significantly worse outcomes after SBRT, and should be considered for clinical trials or treatment intensification.

## Introduction

Stereotactic body radiotherapy (SBRT) for organ-confined prostate cancer has increased in acceptance over the past few years, as multiple studies continue to emerge demonstrating excellent biochemical control and low toxicity with up to 7-year median follow-up (1–9). Low-, intermediate-, and high-risk group stratification is traditionally utilized to assess likelihood of biochemical failure after external beam radiation and identify patients who may benefit from systemic therapy (10). With this approach, intermediate- and high-risk patients are recommended androgen deprivation therapy (ADT) in combination with standard external beam RT to improve outcomes (11). However, it is unclear whether ADT improves outcomes after SBRT (8, 9) as published studies suggest that it may not be of added benefit in the extreme hypofractionation setting. Tumor control mechanisms after high doses per fraction of RT may differ compared to standard fractionated RT, due to a distinct radiobiological effect (12). As a result, it is uncertain whether traditional risk-stratification criteria are reliable measures for predicting outcomes after prostate SBRT. There is a need to explore this further, so that patients can be counseled appropriately about the risks, benefits, and rationale of prostate SBRT compared to other alternatives, and patients predicted to have worse outcomes can be directed toward trials incorporating systemic treatments or treatment intensification.

The purpose of this study was to determine whether traditional risk-stratification criteria remain relevant for predicting outcomes after SBRT. With the longest follow-up to date of any single institution series, we now identify characteristics of patients expected to have excellent outcomes after SBRT vs. those who remain at higher risk of biochemical failure, who may benefit from either treatment intensification and/or systemic therapies.

## Materials and Methods

### Patient Selection

The study cohort was composed of 515 men with biopsy-proven, newly diagnosed non-metastatic prostate cancer, treated as per an IRB-approved protocol between early 2006 and late 2009. Patient characteristics are summarized in Table 1. For the purposes of this analysis, patients were stratified using standard risk categories (11) of low risk (*n* = 324) (PSA <10 and Gleason sum of 6 and clinical stage T1c–T2a); intermediate risk (*n* = 153) (PSA 10–20, Gleason sum of 7 or clinical stage T2b, c); and high risk (*n* = 38) (PSA >20, Gleason 8–10 or T3a–T4). NCCN 1.2016 guidelines suggest further stratification of patients into unfavorable intermediate risk if they have Gleason 4 + 3 = 7 disease, >50% positive cores or >1 intermediate-risk factors; using this definition, there were 106 patients with favorable and 47 patients with unfavorable intermediate-risk disease. Mean patient age at time of treatment was 68 years (range, 43–88). A total of 72 patients received up to 6 months of ADT prior to and during treatment, at the discretion of the urologist. The median PSA at diagnosis was 6.6 ng/mL.

**Table 1:** **Patient characteristics at diagnosis**.

		Entire cohort	
		Number of patients	%
**Age at diagnosis**
	40–49	3	1
	50–59	81	16
	60–69	205	40
	70–79	195	38
	80–89	31	6
	Total	515	
	Mean (range) 68 (43–88)		
**PSA level at treatment**
	Mean (range) 6.6 (0.1–42.9)
	Median	5	
**PSA level at diagnosis**
	<4 ng/mL	200	39
	4–10 ng/mL	251	49
	>10 ng/mL	64	12
**Clinical stage**
	T1a	2	0.3
	T1c	462	89.7
	T2a	51	10
**Gleason score**
	6	357	69
	7 (3 + 4)	84	16
	7 (4 + 3)	42	8
	8 (4 + 4)	24	4.6
	9 (4 + 5)	6	1
	9 (5 + 4)	2	0.4
**Hormone treatment**
	No	443	86
	Yes	72	14
**Dose**	35 Gy	158	31
	36.25 Gy	357	69
**Low risk**	324	63
**Intermediate risk**	153	30
**High risk**	38	7

### Treatment

Fiducial-based image-guided SBRT was delivered using the CyberKnife system (Accuray Inc., Sunnyvale, CA, USA). The treatment specifics of Cyberknife have been published previously (13). General techniques are briefly outlined here. Four gold fiducials were placed in the prostate trans-perineally with ultrasound guidance. This was followed by a non-contrast CT scan in the supine position and in an alpha cradle. Except for those patients that could not undergo an MRI scan, MRI images were obtained and fused into the CT images to better visualize the inferior portion of the prostate. No catheter was used. Anatomical contours of the prostate, seminal vesicles, rectum, bladder, penile bulb, femoral heads, and testes were generated. With homogeneous planning, dose was prescribed to the planning target volume (PTV) that consisted of a volumetric expansion of the prostate by 5 mm, reduced to 3 mm in the posterior direction. During a typical 45-min treatment, fiducial seeds were tracked and adjustments to position were made at 30–60 s intervals. For each morning prior to SBRT, patients underwent a bowel prep including Dulcolax^®^ (Boehringer Ingelheim, Germany) and a Fleet^®^ Enema (C.B. Fleet Company, Inc., Lynchburg, VA, USA). In addition, at least 15–20 min before treatment, all patients received 1500 mg of amifostine (MedImmune, LLC Gaithersburg, MD, USA), mixed in saline, and instilled into the rectum. The dose of radiotherapy consisted of either 35 or 36.25 Gy over five fractions, given daily for all patients.

Dose was normalized to the 83–87% isodose line in order for the prescription dose to cover at least 95% of the PTV. Generally speaking, dose volume histogram (DVH) goals for the rectum were such that the V50% <50% (i.e., the volume receiving 50% of the prescribed dose was <50%), V80% <20%, V90% <10%, and V100% <5%. The bladder DVH goals were V50% <40% and V100% <10%. For the bladder and the rectum, a typical D50 was 40–45% of the maximum dose. The femoral head DVH goal was V40% <5%.

### Follow-up and Toxicity Assessment

The median follow-up for the entire cohort was 84 months (inter-quartile range, 60–96 months). In general, PSAs were obtained at baseline, and prospectively at 3 months post-treatment intervals during the first 2 years and at 6 months intervals thereafter. The PSA relapse definition used was the currently adopted standard of care Phoenix definition (i.e., nadir +2) (14). Biochemical disease-free survival (bDFS or PSA DFS) was calculated with the Kaplan–Meier method and differences between groups determined by the log-rank test. Quality of life (QOL) was assessed using the Expanded Prostate Cancer Index Composite (EPIC) questionnaire (15) at every follow-up visit during the first year and at 24 months. EPIC scores were calculated as defined in Wei et al. (15). In addition, toxicity was assessed using the Radiation Therapy Oncology Group (RTOG) urinary and rectal toxicity scale (16) at every follow-up visit. QOL and toxicity data have been reported previously (7, 8).

For patterns of failure, a prostate biopsy showing positive disease, in the absence of metastatic disease, was counted as a local failure, independent of distant failure status. Biopsy-proven evidence of metastatic disease, or imaging characteristics consistent with widespread metastatic disease attributable to prostate cancer, was counted as distant failure.

### Statistical Analysis

The primary endpoint of the study was bDFS. Kaplan–Meier survival method was used to estimate bDFS and log-rank *p*-values were used to compare the distributions. Cox regression analysis was used to determine whether risk factors were significant after adjusting for competing risks. For Cox regression analysis, the assumption of the proportional hazards model was tested to ensure that these assumptions were not violated. The pretreatment PSA and Gleason score were treated as dichotomous variables, using a cutpoint of 5.4 ng/μL (median value) for PSA and Gleason score <8 vs. ≥8. Two-sided *p* < 0.05 was considered to be statistically significant. The likelihood ratio test was used to determine if there was significant difference in toxicity. JMP Pro 10 (SAS Institute, Cary, NC, USA) was used for statistical analyses.

## Results

### Biochemical Disease-Free Survival

**Figure 1:**
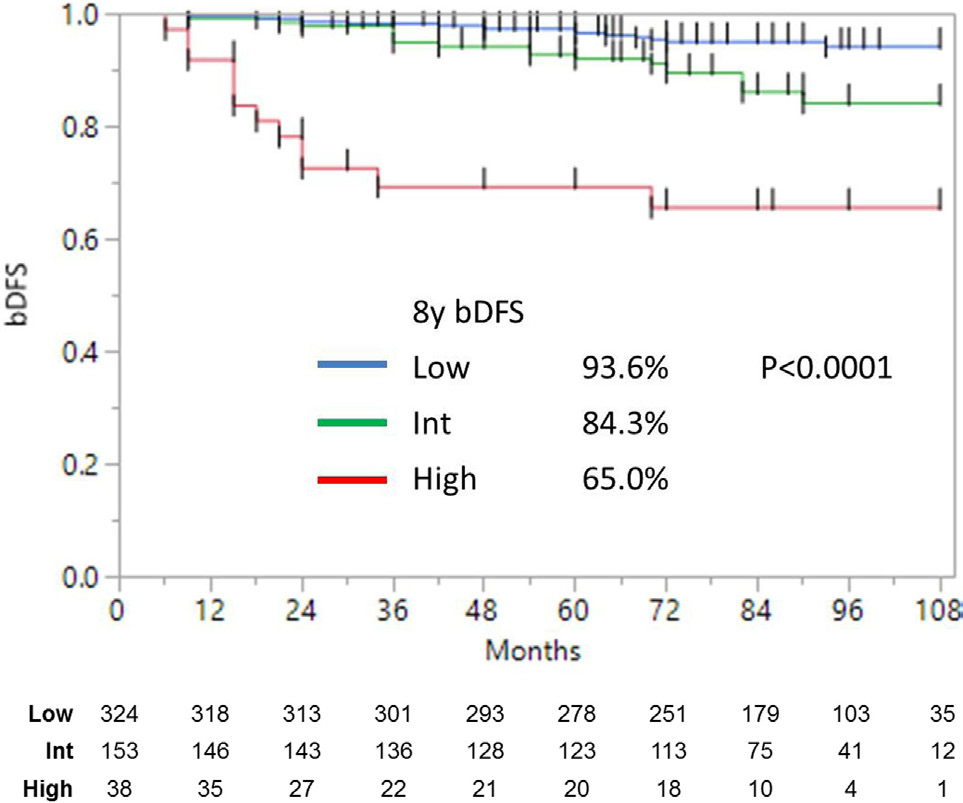
**Biochemical disease-free survival stratified into low-, intermediate-, and high risk (as defined by NCCN)**.

**Figure 2:**
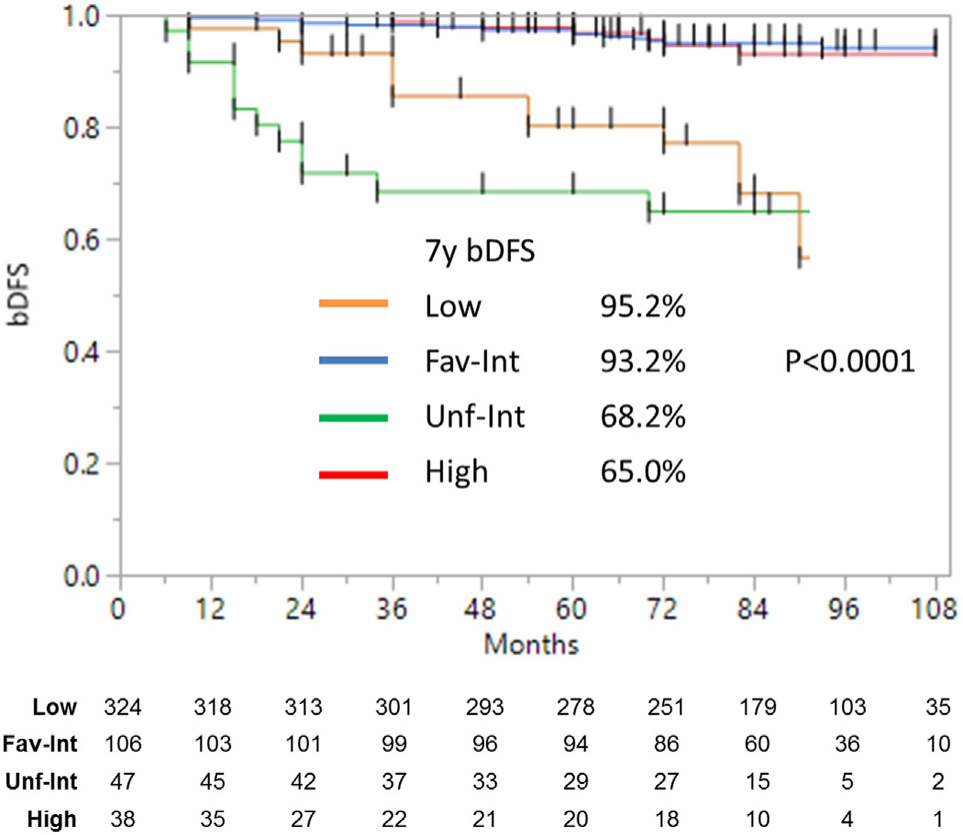
**Biochemical disease-free survival with intermediate-risk group further stratified into favorable vs. unfavorable risk**. There was no significant difference between the low- and favorable intermediate-risk group with follow-up of 9 years. There was a significant (*p* < 0.0001) difference between the low- and favorable intermediate-risk group vs. the unfavorable intermediate-risk and high-risk patients.

Median follow-up was 84 months (range, 6–108 months). At last follow-up, 83 patients were deceased from non-prostate cancer-related causes. Patient characteristics are detailed in Tables 1 and 2. The actuarial 8-year bDFS was 93.6, 84.3, and 65.0% for low-, intermediate-, and high-risk group patients (Figure 1). Per NCCN 1.2016, patients with two or more adverse factors (T2b–T2c, Gleason score = 7, PSA 10–20 ng/mL), >50% positive cores or Gleason 4 + 3 = 7 can be considered as unfavorable intermediate risk. We repeated our analyses, therefore, using these risk-stratification criteria (Figure 2), and found low- and favorable intermediate-risk patients to have similar bDFS (7-year DFS 95.2 vs. 93.2%, respectively, *p* < 0.0001).

**Table 2:** **Patient characteristics of unfavorable-intermediate and high-risk patients at diagnosis**.

		Entire cohort		Unfavorable int		High	
		#	%	#	%	#	%
**Age**
	40–49	2	1.2	1	2.1	0	0
	50–59	5	5.9	2	4.3	3	8
	60–69	26	30.6	13	27.6	13	34
	70–79	38	44.7	24	51.1	14	37
	80–89	15	17.6	7	14.9	8	21
	Total	85		47		38
	Mean (range)	71.1 (43–88)		71 (43–88)		71 (51–86)	
**PSA level at treatment**		
	Mean	10.8 (SD = 8.5)		7.6		14.8			
	Median	8.8		7		10.1	
**PSA level at diagnosis**		
	<4 ng/mL	12	14.1	8	17	4	10.5
	4–10 ng/mL	37	43.5	24	51.1	13	34.2
	>10 ng/mL	36	42.4	15	31.9	21	55.3
**Clinical stage**							
	T1c	66	89.7	38	80.8	28	73.6
	T2a	19	10	9	19.2	10	26.4
**Gleason score**							
	6	3	3.5	0	0	3	7.9
	7 (3 + 4)	8	9.4	7	14.9	1	2.6
	7 (4 + 3)	42	49.4	40	85.1	2	5.3
	8 (4 + 4)	24	28.2	0	0	24	63.2
	9 (4 + 5)	6	7	0	0	6	15.8
	9 (5 + 4)	2	0.2	0	0	2	5.3
**Hormone treatment**							
	No	52	61.1	35	74.5	17	44.7
	Yes	33	38.8	12	25.5	21	55.3
**Dose**	35 Gy	11	12.9	7	15	4	10.5
	36.25 Gy	74	87.1	40	85	34	89.5

**Figure 3:**
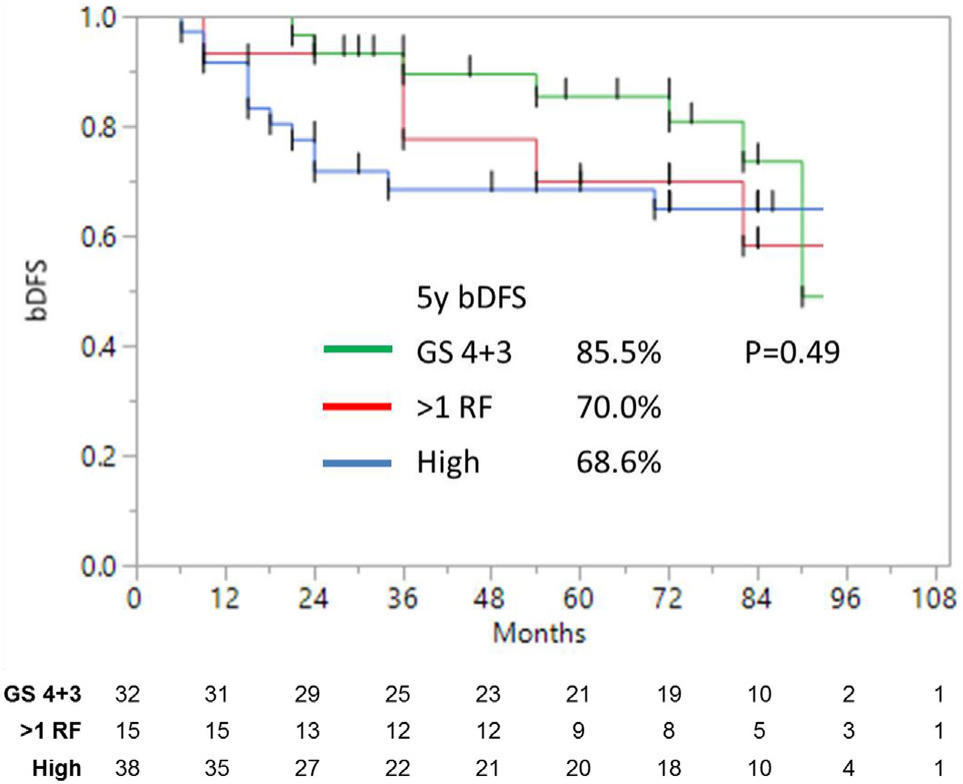
**Biochemical disease-free survival of high-risk and unfavorable intermediate-risk patients with either Gleason 4 + 3 = 7, or >1 risk factors, shows no significant difference**.

Unfavorable intermediate-risk patients performed significantly worse, with outcomes similar to high-risk patients (7-year DFS 68.2 vs. 65.0%, respectively; *p* < 0.0001). To analyze this further (Figure 3), unfavorable intermediate-risk patients with Gleason 4 + 3 = 7 or >1 intermediate-risk factor were compared to high-risk patients, and no significant differences were noted (5-year DFS 85.5, 70.0, and 68.6%, respectively; *p* = 0.49).

### SBRT Dose Does Not Impact Biochemical Disease-Free Survival

**Figure 4:**
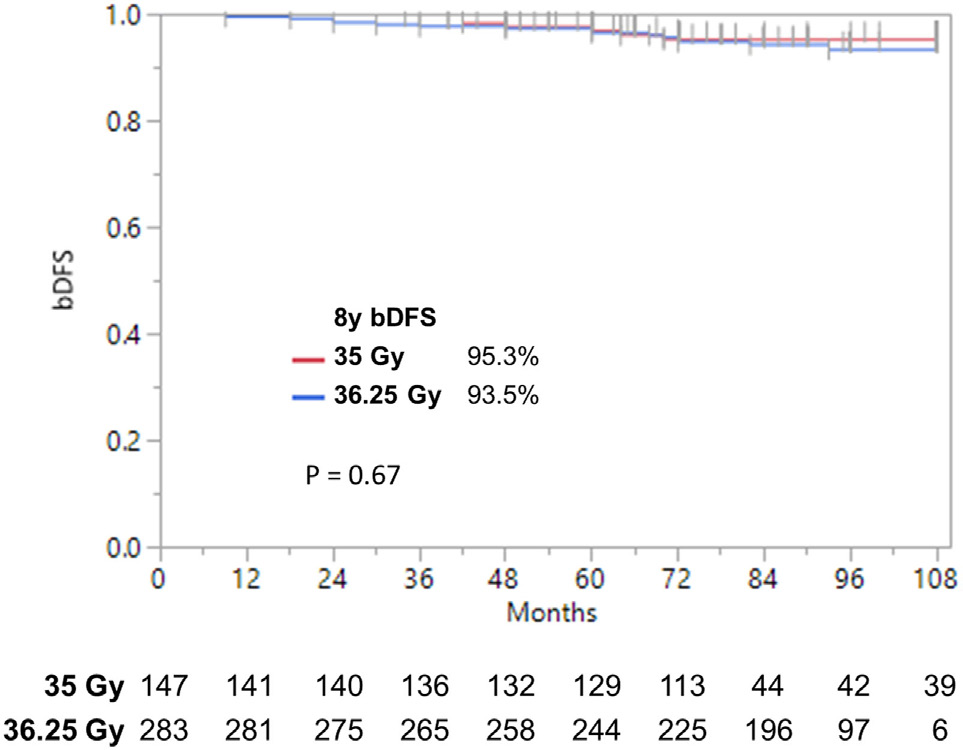
**Biochemical disease-free survival stratified by dose**. bDFS in low- and favorable intermediate-risk patients. With follow-up up to 9 years, we find no significant difference by dose.

Patients were treated to either 35 or 36.25 Gy in five consecutive daily fractions. Given that favorable intermediate-risk patients performed as well as low-risk patients, we combined both groups prior to performing our analysis and found no significant difference in biochemical outcome as a function of SBRT dose (Figure 4) (9-year DFS 95.3 vs. 93.5% for 35 vs. 36.25 Gy, *p* = 0.67). A similar analysis was performed for unfavorable intermediate-risk and high-risk patients, and again we found no difference in outcome as a function of SBRT dose.

### Risk Factors for Biochemical Failure after Prostate SBRT

Results of Cox multivariable regression analysis are shown in Table 3. Pretreatment factors included in the analyses were use of ADT, baseline PSA, Gleason score, and dose. The only variable found to be significant as a predictor for biochemical failure was Gleason score, which is a known prognostic factor (*p* < 0.0001). Use of ADT in the unfavorable-intermediate and high-risk patient was analyzed separately, and there was no statistically significant difference (*p* > 0.05).

**Table 3:** **Relative risk and *p*-value from Cox regression multivariable analysis for pretreatment predictors of biochemical failure**.

Pretreatment variable	Risk (95% CI)	*p*-Value
Hormones (no vs. yes)	1.08 (0.48–2.29)	0.84
Dose (35 vs. 36.25 Gy)	1.53 (0.74–3.6)	0.26
Gleason score (≤7, ≥8)	6.34 (2.74–13.83)	<0.0001
PSA (≤5.4 ng/ul, >5.4 ng/ul)	1.45 (0.79–2.70)	0.22

*CI, confidence interval; Gy, Gray*.

### Patterns of Failure after Prostate SBRT

**Figure 5:**
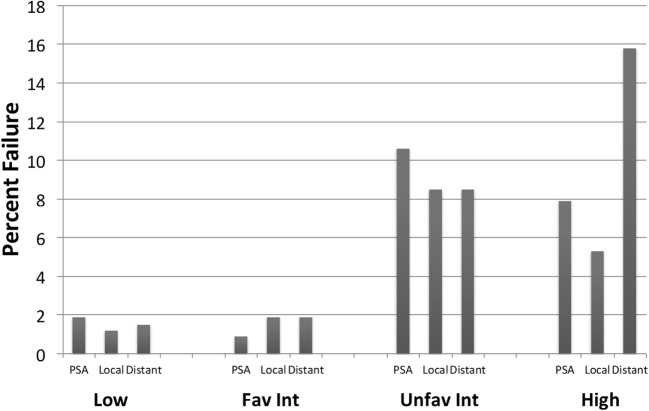
**Failure patterns after prostate SBRT: type of failure**. % PSA, local and distant failure stratified by risk group. All cases of local failure were proven on biopsy. Distant failure was biopsy proven, unless widespread metastatic disease on imaging.

**Figure 6:**
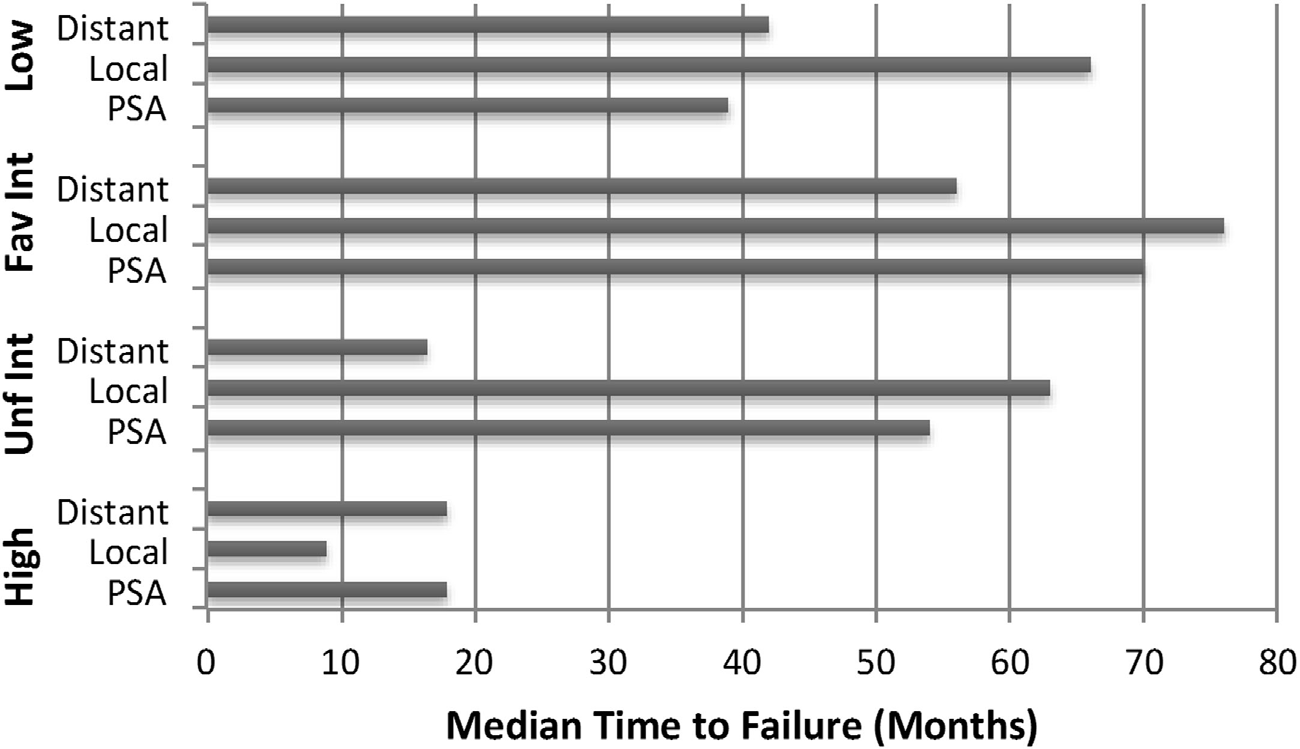
**Failure patterns after prostate SBRT: time to failure**. Median time to PSA, local and distant failure subdivided by risk group.

Crude percentages of PSA, local and distant failure for low, favorable intermediate-risk, unfavorable intermediate-risk, and high-risk patients are shown in Figure 5. The median time to each type of failure is graphically depicted in Figure 6. Patients in the unfavorable intermediate-risk and high-risk category had a shorter median time to distant failure compared to patients in the low- and favorable intermediate-risk category (15.5 and 18 months vs. 42 and 55 months, respectively, *p* < 0.05). Patients in the high-risk category had a significantly shorter median time to biochemical (18 vs. 51 months) and local failure (9 vs. 66 months) compared to the other risk groups.

## Discussion

Our study, with the longest follow-up to date of any single institution series that has been reported, suggests that traditional risk-stratification criteria can be simplified to predict outcomes after SBRT. Patients with Gleason score of 3 + 4 with PSA <10, or Gleason 3 + 3 with PSA 10–20, are historically classified as intermediate-risk. However, in our dataset with up to 9 years of follow-up, we find no significant difference in bDFS between this favorable intermediate-risk group and low-risk patients. Both local and distant relapse rates remain ≤2% after a dose to 35 or 36.25 Gy in five fractions, suggesting that further dose escalation or systemic treatment will not be of significant benefit. This brings into question ongoing dose-escalation protocols for low and intermediate-risk prostate cancer patients, which may be needlessly placing patients at risk of higher toxicity without providing meaningful local control benefit.

In this study, unfavorable intermediate-risk patients fared no better than high-risk patients. On multivariate analysis, Gleason score was the only factor significant for bDFS, suggesting that primary Gleason score is a driving force behind prostate cancer outcomes after SBRT. This is consistent with multiple studies demonstrating the relevance of Gleason score as a prognostic factor across all treatments for prostate cancer (17, 18). Use of ADT or higher dose did not have a significant impact on outcomes, consistent with emerging literature, suggesting that ADT may be of limited benefit in combination with SBRT (8). Consistently, a BED equivalent of 200 Gy (equivalent of 35–36.25 Gy assuming α/β of 1.5) has been suggested as the maximum dose beyond which there are no improvements in disease control, but only added toxicity (19).

Our results for intermediate- and high-risk patients are comparable to those achieved with IMRT and other modalities, and support use of SBRT as a treatment alternative in this patient population. In our study, the predominant pattern of failure is biochemical and/or distant. As such, PSA failures likely represent undetectable micrometastatic disease already present at the time of diagnosis and initial treatment. Given this scenario, dose escalation to the prostate alone is unlikely to be of substantial benefit, while a combination strategy incorporating systemic treatment into the SBRT treatment paradigm may be better suited to combat distant spread. Hopefully, future trials with novel treatment strategies will focus on this subgroup of patients.

Several limitations of our study include lack of information on % positive cores, relatively small numbers for high-risk patients, and the single institution nature of the trial. Also, the use of ADT in a subset of patients at the urologist’s discretion was not found to be significant for bDFS, but nevertheless complicates the analysis. Despite these limitations, given that prostate SBRT is a newly emerging technique with minimal data on long-term outcomes, we believe our present work will be hypothesis generating and of use to radiation oncologists, until data from larger prospective studies become available.

In summary, SBRT to well-tolerated total doses of 35–36.35 Gy in five fractions continues to show excellent biochemical and local control with up to 9 years of follow-up. A significant bDFS difference exists for favorable vs. unfavorable intermediate-risk groups, suggesting that a simple risk-stratification construct of lower vs. higher-risk can be utilized to discuss risks of failure when patients consider prostate SBRT, until data from current prospective studies mature. While still requiring confirmation by additional prospective trials, patients with Gleason score of 3 + 4 with PSA <10, or Gleason 3 + 3 with PSA 10–20 who would traditionally have been classified as intermediate risk, had excellent outcomes in this study, no different from low-risk patients, and may not require dose escalation or consideration of ADT.

The remaining challenge is how to improve outcomes for patients with unfavorable intermediate-risk and high-risk disease, who remain at high risk of distant failure. In this subset, combination treatment or novel therapies are warranted for clinical trials testing. To our knowledge, this series provides clinical results with the longest follow-up reported, supporting existing guidelines that incorporate prostate SBRT into the treatment paradigm for low- and intermediate-risk patients.

## Author Contributions

AK wrote the manuscript, and helped design data analysis and perform analysis. SF helped write the manuscript and comment on analysis of data. JK wrote the manuscript, and helped design data analysis and perform analysis.

## Conflict of Interest Statement

The authors declare that the research was conducted in the absence of any commercial or financial relationships that could be construed as a potential conflict of interest.
